# The research landscape of ferroptosis in ischemic stroke: a bibliometric analysis of the literature from 2017 to 2025 based on the WOS and PubMed databases

**DOI:** 10.3389/fncel.2026.1836830

**Published:** 2026-08-26

**Authors:** Hongmei Tang, Yujie Zhang, Mingxia Zhang, Jia Hu, Lingxue Wang, Shuangyang Li, Yuting Pu, Xue Bai

**Affiliations:** 1 Department of Neurology, The Affiliated Traditional Chinese Medicine Hospital of Southwest Medical University, Luzhou, Sichuan, China; 2 School of Integrated Traditional Chinese and Western Medicine, Southwest Medical University, Luzhou, Sichuan, China

**Keywords:** bibliometric analysis, CiteSpace, ferroptosis, ischemic stroke, knowledge map, research hotspots, research trajectory, VOSviewer

## Abstract

**Background:**

Ischemic stroke is a leading cause of disability and mortality worldwide. Ferroptosis—an iron-dependent, lipid peroxidation-driven programmed cell death—has emerged as a key contributor to post-stroke neuronal injury, with research expanding rapidly since 2017.

**Objective:**

This study employs bibliometric methods to systematically analyze literature on ferroptosis in ischemic stroke from 2017 to 2025, aiming to elucidate the field’s development trajectory, research hotspots, emerging frontiers, and collaborative networks.

**Methods:**

Data were retrieved from the Web of Science Core Collection and PubMed, limited to English articles and reviews published between 2017 and 2025. For PubMed, an additional search was conducted to capture empirical studies on ferroptosis inhibitors. Bibliometric analyses were performed using VOSviewer, CiteSpace, and Bibliometrix R package.

**Results:**

A total of 646 WoS-indexed articles were analyzed. Annual publication output showed exponential growth. China, the United States, and India led in publication volume, with China accounting for over half of global output; international collaboration centered on China–US partnerships. Research hotspots focused on molecular mechanisms (GPX4, System Xc^−^, Nrf2/HO-1), key regulators (ACSL4, FSP1), and therapeutic strategies (ferroptosis inhibitors, natural compounds). Analysis of 64 PubMed-indexed studies on ferroptosis inhibitors revealed that China also dominated this area, with the National Natural Science Foundation of China as the primary funding source. Emerging frontiers indicate a shift from mechanistic studies toward clinical translation, including biomarker development, nano-targeted delivery, and neurovascular unit protection.

**Conclusion:**

From 2017 to 2025, research on ferroptosis in ischemic stroke has rapidly evolved from concept introduction and mechanistic exploration toward clinical translation. Bibliometric analysis delineates a clear trajectory and evolving knowledge framework. Future research will likely emphasize interdisciplinary integration, clinical translation, and personalized therapeutic strategies.

## 1 Introduction

Ischemic stroke is an acute cerebrovascular disease caused by an interruption in the blood supply to the brain (Correa-Paz et al., 2022), leading to ischemia and hypoxic necrosis of brain tissue (Cheng et al., 2026), which in turn results in neurological deficits. It accounts for 70–80% of all stroke types (Yoshitomi and Nagasaki, 2022). At the same time, studies indicate that risk factors such as hypertension (Diener and Hankey, 2020), hyperglycemia, smoking, and alcohol consumption (Im et al., 2023) are contributing to a year-on-year increase in the incidence of ischemic stroke (An et al., 2025). Although reperfusion therapies (such as intravenous thrombolysis and mechanical thrombectomy) are key methods for restoring blood flow (Lin et al., 2025), they have a narrow therapeutic time window and may be accompanied by life-threatening reperfusion injury (Wang et al., 2025). Therefore, conducting in-depth research into the complex mechanisms of neuronal cell death following stroke, identifying new neuroprotective targets, and exploring effective neuroprotective strategies represent major scientific challenges in reducing mortality and disability rates.

Programmed cell death (PCD) is a central pathological process in ischemic brain injury (Qin et al., 2025). In addition to the classical forms of apoptosis, necroptosis, and autophagy, Dixon et al. first proposed and named a novel form of iron-dependent, lipid peroxidation-driven programmed cell death—ferroptosis—in 2012 (Dixon et al., 2012). Its core features include glutathione (GSH) depletion (de Pinto et al., 2006), inhibition of glutathione peroxidase 4 (GPX4) activity, and uncontrolled accumulation of lipid reactive oxygen species (Lipid-ROS) (Dixon et al., 2012; Stockwell et al., 2017). A growing body of evidence indicates that the ferroptosis signaling pathway is significantly activated during the acute phase of ischemic stroke; inhibiting ferroptosis can effectively reduce infarct volume and improve neurological outcomes (Li et al., 2024), making it a highly promising new therapeutic target.

Since 2017, a large number of studies on the role of iron death in ischemic stroke have emerged and continued to be a hot topic, forming a multidisciplinary research field. Faced with a vast and rapidly growing body of literature, researchers are confronted with the practical challenge of how to systematically and objectively grasp the full picture of developments in this field, identify key contributors, gain insights into the evolution of knowledge structures, and predict future trends. Bibliometrics, as an interdisciplinary field that employs mathematical and statistical methods to conduct quantitative analysis of bibliographic data, can effectively reveal the developmental dynamics, research hotspots, and cutting-edge trends within a specific discipline (Ninkov et al., 2022).

A prior bibliometric study by Chen et al. (2021) systematically examined the literature on ferroptosis in stroke (including both ischemic and hemorrhagic types) using the Web of Science database, covering publications up to 2021. That study identified early research hotspots and noted a lack of international collaboration among Chinese groups at that time. This earlier work has been recognized as an important early mapping in the field (Gao et al., 2023).

However, our study focuses specifically on ischemic stroke, the most common subtype of stroke, which has distinct pathophysiological mechanisms and therapeutic targets compared to hemorrhagic stroke. Moreover, the field has experienced explosive growth since 2020, with a surge of studies focusing on specific ferroptosis inhibitors and preclinical ischemic stroke models. No prior bibliometric work has specifically analyzed inhibitor-focused empirical studies from PubMed or integrated data through 2025, nor has any focused exclusively on the ischemic subtype.

Therefore, building upon the earlier work by Chen et al. (2021), the present study extends the temporal coverage to 2025, refines the disease scope to ischemic stroke, and incorporates a separate analysis of inhibitor-focused studies from PubMed. By constructing a scientific knowledge map, this paper seeks to illustrate the temporal evolution of research output in this field, assess the contributions and collaborative networks of countries, institutions, and authors, and highlight the formation and evolution of the field’s knowledge base, research hotspots, and themes, as well as current research frontiers and future directions.

This study will provide a clear academic map for researchers new to the field and offer strategic guidance and data support for seasoned researchers.

## 2 Research methods

### 2.1 Data collection and retrieval strategy

To ensure the authority and high quality of the literature data, this study selected the Web of Science Core Collection and the PubMed database as data sources. All retrieval operations were performed on January 1, 2026. The 2025 data represents the actual number of records indexed in the database on that day. There may be a very small number of cases where records were included with a slight delay, but this does not affect the main conclusions.

#### 2.1.1 Web of Science search

Data were retrieved from the Web of Science Core Collection (including SCI-Expanded, SSCI, and A&HCI indexes). A subject term (TS) search was employed, with the following search query: TS = ((“ischemic stroke” OR “brain ischemia” OR “cerebral ischemia” OR “acute ischemic stroke” OR “middle cerebral artery occlusion”) AND (“ferroptosis” OR “ferroptotic”)). The document types were restricted to “Article” and “Review,” the language was restricted to English, and the time span was from January 1, 2017, to December 31, 2025. The starting year of 2017 was chosen because the first study directly linking ferroptosis to ischemic stroke was published in that year (Tuo et al., 2017); prior to 2017, relevant publications were too few to support reliable trend analysis.

#### 2.1.2 PubMed search

To focus on empirical studies of ferroptosis inhibitors, a more refined search query was used in PubMed to identify relevant animal experiments and preliminary clinical studies. The search query combined subject headings and free-text terms, as follows: ((“ferroptosis inhibitors” [MeSH Terms] OR “ferroptosis” [MeSH Terms] OR ferroptosis [Title/Abstract]) AND (deferoxamine [Title/Abstract] OR deferiprone [Title/Abstract] OR “iron chelator” [Title/Abstract] OR ferrostatin-1 [Title/Abstract] OR liproxstatin-1[Title/Abstract]) OR “ferroptosis inhibitor” [Title/Abstract]) AND (“ischemic stroke” [MeSH Terms] OR “brain ischemia” [MeSH Terms] OR “ischemic stroke”[Title/Abstract] OR “cerebral ischemia” [Title/Abstract] OR “middle cerebral artery occlusion” [Title/Abstract] OR MCAO[Title/Abstract]). The document type was restricted to “Article,” and the language and time range were consistent with the WoS search.

### 2.2 Data cleaning and processing

The search results were exported in “plain text file” format as full records and cited references. Record content included “author, title, source publication, abstract, author keywords, Keywords Plus, and cited references.” Software such as the Bibliometrix R package, VOSviewer, and CiteSpace was used to merge and deduplicate the data. Irrelevant documents, such as conference abstracts, editorials, and correction notices, were manually excluded, ultimately yielding a clean dataset of literature for analysis.

### 2.3 Analysis tools and process

This study employed multiple bibliometric software tools for comprehensive analysis: the Bibliometrix R package was used for descriptive statistical analysis, including annual publication volumes and the distribution of output by country/region, institution, author, and journal; VOSviewer (version 1.6.20) was used to construct visual network maps, focusing on the analysis of country/institutional collaboration networks, author collaboration networks, and keyword co-occurrence analysis. VOSviewer presents results through visual clustering, where node size represents frequency or weight, line thickness represents the strength of collaboration or co-occurrence, and color represents different clusters; CiteSpace (version 6.3. R1): Used for co-citation analysis (to reveal the knowledge base), keyword burst detection (to identify research frontiers), and timezone views (to illustrate the evolution of research topics).

The analysis process primarily includes: (1) First, based on the analysis and retrieval results from WOSCC and PubMed, conduct a preliminary analysis of general literature information, including publication year, country, organization, journal, and authors. Perform descriptive statistics to present a macro-level overview of the field; (2) Citation analysis: identify the knowledge base and classic literature; (3) Collaboration network analysis: identify core research forces; (4) Keyword analysis: reveal research hotspots and frontier trends. Because the two searches had different objectives and scopes, their results are not directly comparable; therefore, all analyses in this paper are presented separately.

## 3 Results

**Figure 1:**
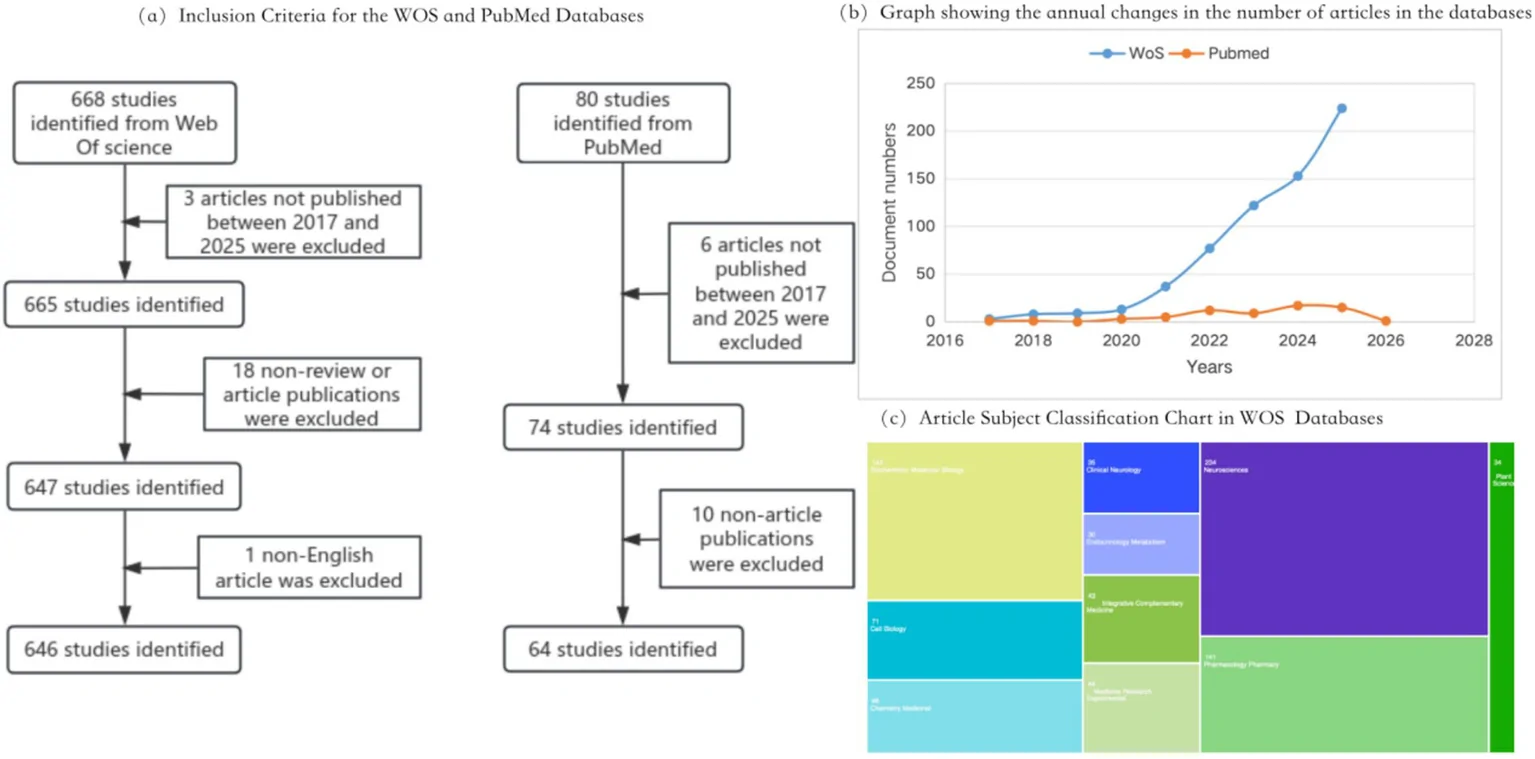
**(a)** Flowchart of the literature search and screening process for the WOS and PubMed databases. **(b)** Line graph showing the annual growth in the number of articles in the WOS and PubMed databases. **(c)** Diagram of the disciplinary classification of articles in the WOS database.

A total of 646 valid articles from the WOS database and 64 valid articles from the PubMed database were included in the analysis. The literature search and screening process is shown in Figure 1a.

### 3.1 Annual publication trends

Figure 1b shows the annual publication volume of global research on ischemic stroke and ferroptosis in the WOS database from 2017 to 2025. Starting from single digits in 2017, the growth rate accelerated after 2020, exhibiting near-exponential growth from 2023 to 2025. This explosive growth in publication volume (with an estimated compound annual growth rate (CAGR) exceeding 60%) strongly indicates that this field is in the rapid growth phase of its lifecycle, attracting increasing attention and investment from research teams worldwide, and represents a dynamic frontier of research. In the PubMed database, the annual number of publications on empirical studies of ferroptosis inhibitors in ischemic stroke shows an upward trend. This indicates that research on ferroptosis inhibitors in ischemic stroke is shifting from basic mechanism exploration toward deeper application, marking a golden period for research output. It is an important direction that cannot be overlooked in future research on stroke treatment strategies. The disciplinary classification of research literature on ferroptosis in ischemic stroke retrieved from the WOS database is shown in Figure 1c, indicating that research in this field is currently concentrated in basic science. Future efforts should focus more on translating these findings into clinical applications.

### 3.2 Distribution by country/region and institution

**Figure 2:**
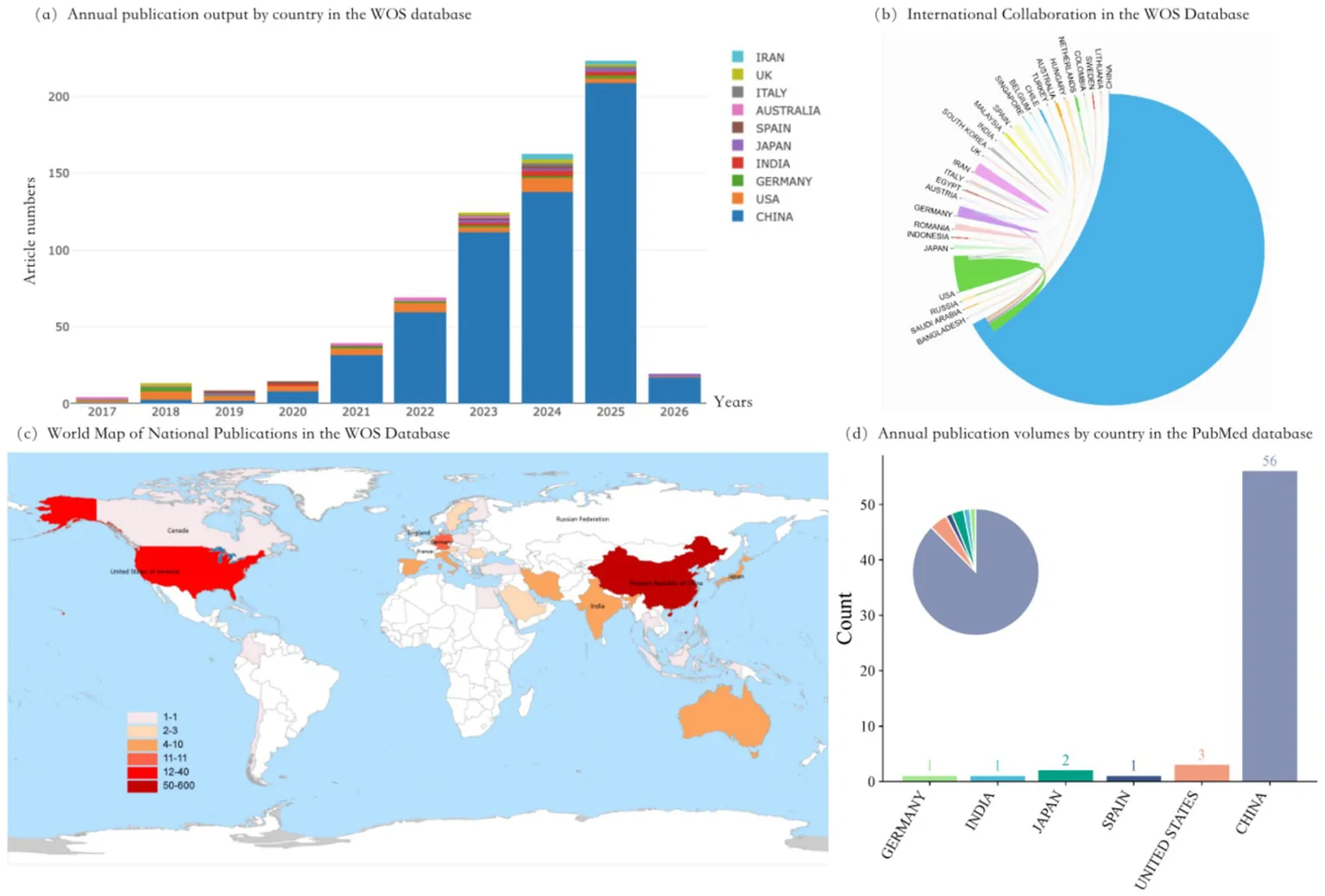
**(a)** Annual publication output by country in the WOS database. **(b)** Inter-country collaboration in the WOS database. **(c)** World map of publication output by country in the WOS database. **(d)** Annual publication output by country in the PubMed database.

The height of the bar chart, the area of the chord diagram, and the color of the nodes represent the number of publications. Results from the WOS database indicate that China has the highest publication output, far exceeding that of other countries, followed by the United States, Germany, India, Japan, Australia, and Spain, as shown in Figure 2a. The connections in the network diagram reveal that collaboration between China and the United States is the closest, forming a central axis. There is also extensive collaboration among European countries and with the United States, as shown in Figure 2b. China and the United States are the absolute leaders in this field, as shown in Figure 2c. An analysis of national publication volumes based on the PubMed database (Figure 2d) reveals that China holds an absolute dominant position in the field of ferroptosis inhibitors and ischemic stroke research, with a publication volume of 56 papers, far outpacing other countries. The United States ranks second with 3 papers, followed by Japan with 2, and Germany, India, and Spain with 1 each. This indicates that this research direction is currently driven primarily by Chinese research teams, while other countries are still in the early stages or playing a catch-up role, and the research landscape exhibits distinct regional concentration.

**Figure 3:**
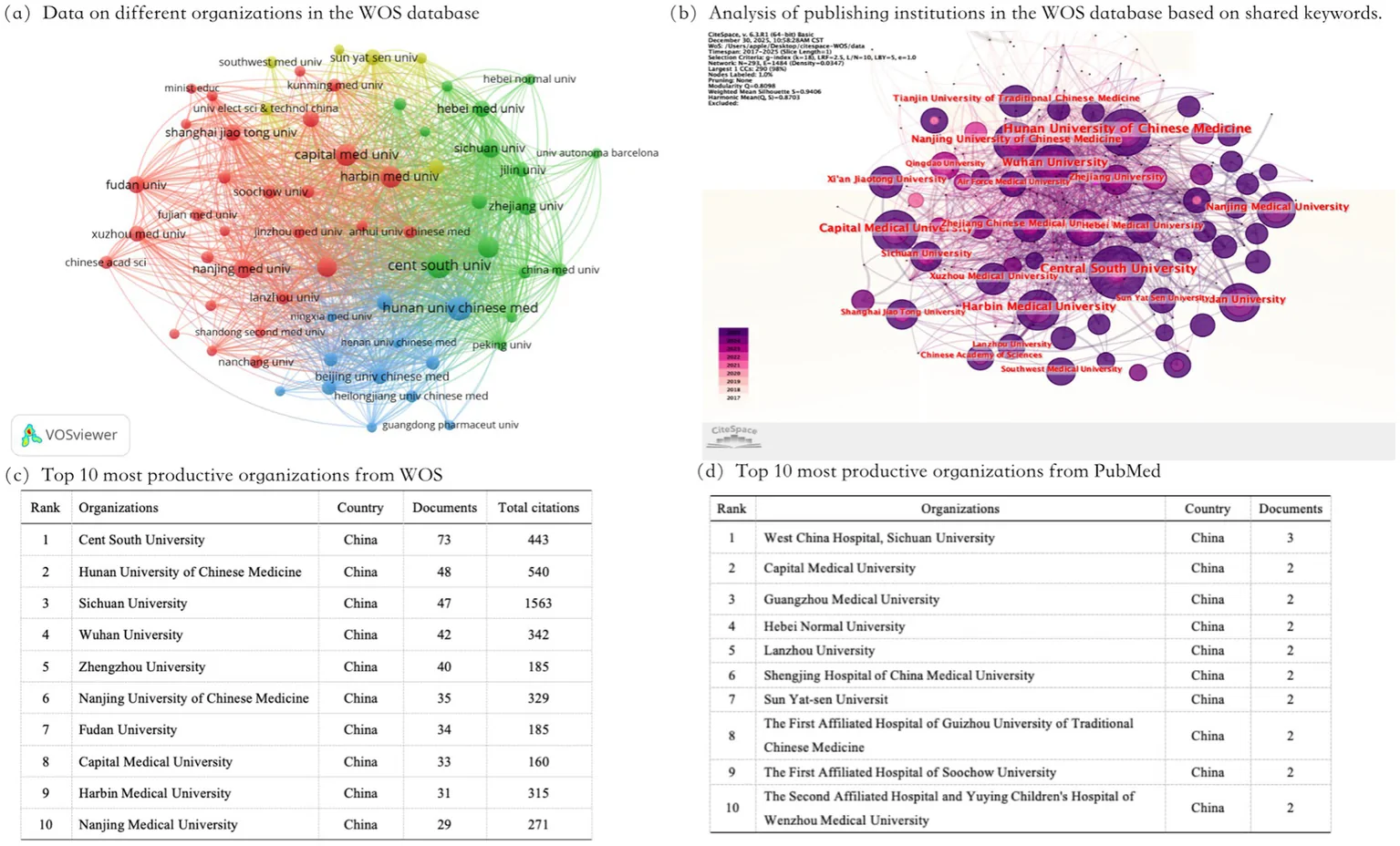
**(a)** Network diagram of institutions publishing in the WOS database. **(b)** Analysis of publishing institutions in the WOS database based on shared keywords. Nodes represent institutions; edges indicate shared keywords between institutions, with node size proportional to publication count. **(c)** Diagram of the top 10 institutions by publication volume in the WOS database **(d)** Diagram of the top 10 institutions by publication volume in the PubMed database.

Institutional analysis shows that Chinese institutions such as Central South University, Hunan University of Traditional Chinese Medicine, Sichuan University, Wuhan University, and Zhengzhou University lead in publication output, as shown in Figures 3a–c. Leading international institutions include the University of Autonoma Barcelona, Massachusetts General Hospital, and the University of Melbourne. The collaboration network exhibits a distinct pattern characterized by “close domestic collaboration and international collaboration primarily involving partnerships between top-tier institutions.” Based on the PubMed database, the top five institutions by publication volume are, in order: West China Hospital of Sichuan University, Capital Medical University, Guangzhou Medical University, Hubei Normal University, and Lanzhou University. The data is derived from PubMed analysis results, and the specific distribution is shown in Figure 3d.

### 3.3 Distribution of authors and journals

**Figure 4:**
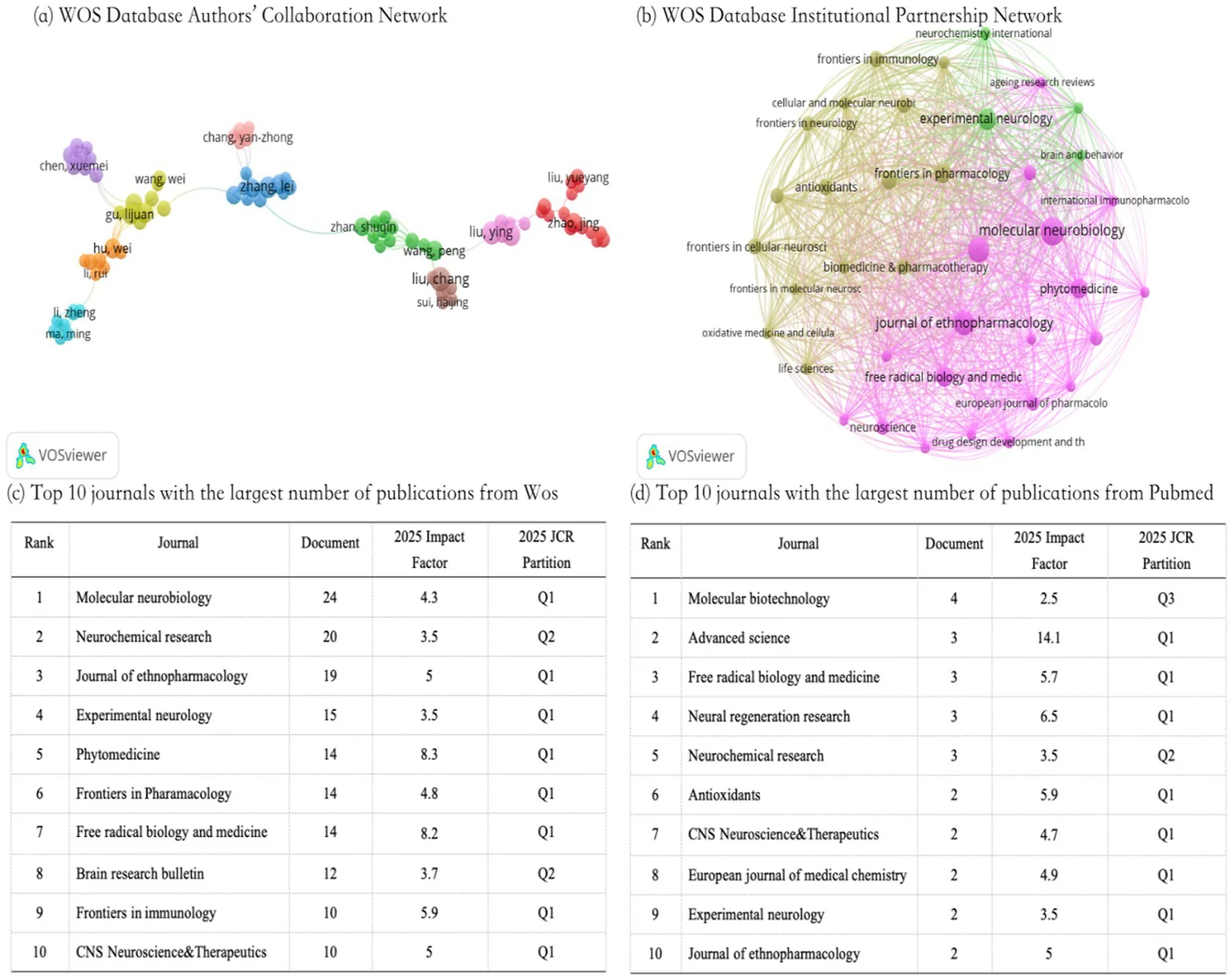
**(a)** Author collaboration network in the WOS database. **(b)** Publication network of journals in the WOS database. **(c)** The top 10 journals by number of articles in the WOS database. **(d)** The top 10 journals by number of articles in the PubMed database.

By analyzing the author collaboration network, it is possible to identify the core group of authors in this field. Based on the Web of Science (WOS) database, high-output Chinese authors include Peng Lei, Zhigang Mei and Ge Jinwen as shown in Figure 4a and Table 1. Peng Lei’s researches focus on the pathogenesis of Alzheimer’s disease and the molecular mechanisms of ferroptosis (Guo et al., 2023). Zhigang Mei, who specializes in the mechanisms of integrated traditional Chinese and Western medicine for the prevention and treatment of brain diseases such as stroke, neurodegenerative diseases, and inflammatory diseases such as autoimmune disorders (Guo et al., 2021). And Ge Jinwen,whose research focuses on the prevention and treatment of cerebrovascular diseases and diabetic vascular complications using integrated traditional Chinese and Western medicine (Zhong et al., 2025). Statistical data indicate that representative foreign authors include Núria DeGregorio-Rocasolano (whose research focuses on the cellular and molecular mechanisms of neurodegenerative diseases, particularly Alzheimer’s disease and the pathological processes of excitotoxic neurodeath), Teresa Gasull (whose research focuses on cerebral ischemia and iron metabolism, as well as stroke biomarkers), and Octavi Martí-Sistac (who primarily investigates the pathological mechanisms and potential therapeutic strategies for brain injury and neurological diseases, with a particular focus on the role of iron metabolism imbalance in stroke, cerebral ischemia, and intracerebral hemorrhage). The network map reveals multiple closely collaborating author teams, forming distinct academic communities.

**Table 1:** Top 10 active authors with most documents from WOS.

Rank	Authors	Organizations	Documents	Total citations
1	Lei Peng	Sichuan University	9	1,502
2	Liu Chang	Jinzhou Medical University	9	154
3	Tuo Qingzhang	West China Hospital, Sichuan University	8	1,456
4	Liu Ying	Central South University	8	249
5	Ge Jinwen	Hunan University of Chinese Medicine	8	448
6	Zhao, Jie	Central South University	7	300
7	Mei, Zhigang	Hunan University of Chinese Medicine	7	361
8	Zhang Lei	the Second Affiliated Hospital of Xi’an Jiaotong University	7	168
9	Zhang Wei	Capital Medical University	7	44
10	Zhao Jing	Hunan University of Chinese Medicine	6	384

As shown in Figure 4b, research findings in this field from journals indexed in the Web of Science database are primarily published in authoritative journals spanning interdisciplinary fields such as neuroscience, molecular biology, pharmacology, and cell biology. Representative journals include classic neuroscience journals such as Molecular Neurobiology, Neurochemical Research, and Experimental Neurology, as well as mainstream publications in the field of pharmacology such as Journal of Ethnopharmacology, Phytomedicine, and Frontiers in Pharmacology. It is worth noting that although the impact factors of these journals are mostly in the mid-range, they demonstrate outstanding professional recognition and academic leadership in their respective fields, indirectly confirming that research findings in this field possess solid scientific value and stable academic influence.

Analysis based on the PubMed database shows that the top 10 journals with the highest number of published papers are Molecular Biotechnology, Advanced Science, Free Radical Biology and Medicine, Neural Regeneration Research, Neurochemical Research, Antioxidants, CNS Neuroscience and Therapeutics, European Journal of Medicinal Chemistry, Experimental Neurology, and Journal of Ethnopharmacology, as shown in Figure 4c and Table 2. High-output authors include Wei Zhang (whose research focuses on ultrasound diagnosis of Parkinson’s disease, assessment of carotid artery lesions, tumor ultrasound ablation, and super-resolution ultrasound imaging), Anbang Zhang (who focuses on integrated traditional Chinese and Western medicine rehabilitation for post-stroke sequelae such as spasticity and dysphagia), Bo Pang (whose research centers on neurological rehabilitation, with a focus on integrated traditional Chinese and Western medicine rehabilitation for hemiplegia following cerebral infarction).

**Table 2:** Top 10 active authors with most documents from PubMed.

Rank	Authors	Organizations	Documents
1	Zhang Wei	Beijing Tiantan Hospital, Capital Medical University	3
2	Zhang Anbang	The First Affiliated Hospital of Guizhou University of Traditional Chinese Medicine	2
3	Pang Bo	The First Affiliated Hospital of Guizhou University of Traditional Chinese Medicine	2
4	Zhang Guilian	The Second Affiliated Hospital of Xi’an Jiaotong University	2
5	Zhou Huihao	Research Center for Drug Discovery, School of Pharmaceutical Sciences, Sun Yat-sen University	2
6	Lu Jialiang	The Second Affiliated Hospital of Xi’an Jiaotong University	2
7	Zhao Jie	Affiliated Zhongshan Hospital of Dalian University	2
8	Zhao Jing	Minhang Hospital, Fudan University	2
9	Jun Xu	Research Center for Drug Discovery, School of Pharmaceutical Sciences, Sun Yat-sen University	2
10	Zhang Min	The Second School of Medicine, The Second Affiliated Hospital and Yuying Children’s Hospital of Wenzhou Medical University	2

### 3.4 Funding

According to funding data from the Web of Science (WOS) database (Table 3), China plays an absolutely dominant role in the funding support system for this research field. Among the top 10 funding sources, Chinese funding programs accounted for 9 spots, with a combined frequency of 268. Among these, the National Natural Science Foundation of China (NSFC) tops the list with 186 mentions, serving as the core pillar of research in this field. Meanwhile, the China Postdoctoral Science Foundation, the National Key Research and Development Program, and local science funds from regions such as Hunan and Beijing collectively form a multi-tiered funding network spanning from basic research to the cultivation of young talent, and from the national to the regional level. In contrast, international funding sources are relatively limited. The U. S. National Institutes of Health (NIH) is the only non-Chinese funding agency in the top 10, with 6 mentions, while the European Union ranks third with 15 mentions; however, the gap in funding scale compared to China is significant. This funding landscape underscores the substantial research funding behind China’s rise in this field. The sustained, high-intensity support from national-level funds, exemplified by the NSFC, has laid a solid financial foundation for China’s flourishing development in this cutting-edge field.

**Table 3:** The top 10 funding sources from WOS.

Rank	Funding source	Frequency
1	National Natural Science Foundation of China (NSFC)	186
2	Natural Science Foundation of China	25
3	European Union	15
4	Natural Science Foundation of Hunan Province	12
5	China Postdoctoral Science Foundation	10
6	Fundamental Research Funds for the Central Universities	9
7	National Key Research and Development Program of China	9
8	National Institutes of Health (NIH)	6
9	National Key R&D Program of China	6
10	Beijing Natural Science Foundation	5

### 3.5 Co-citation analysis and knowledge base analysis

**Figure 5:**
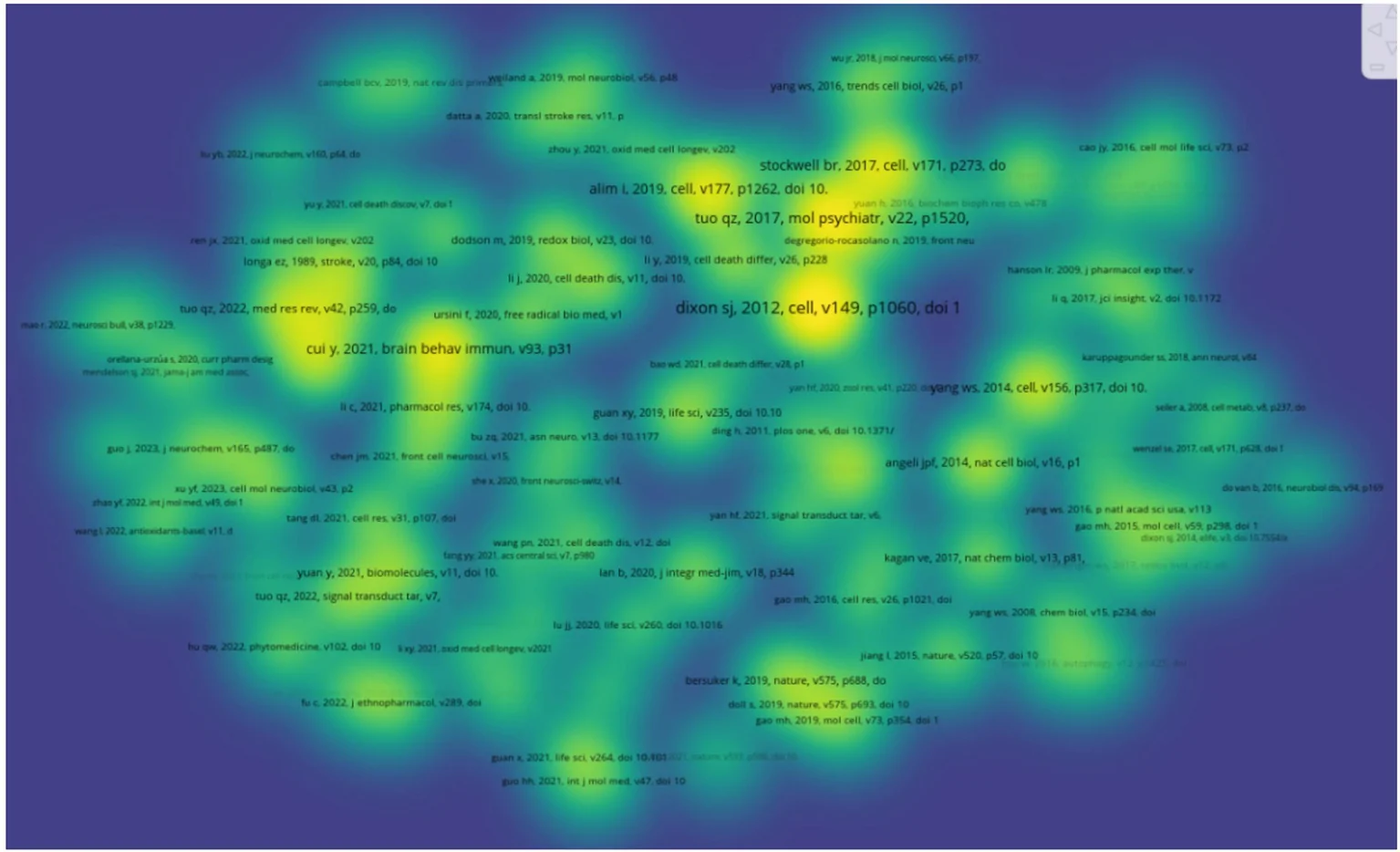
Highly cited references in the WOS database.

A cluster analysis of highly cited references from the WOS database was conducted using VosViewer, as shown in Figure 5. These highly cited references form the theoretical foundation of the field and guide the direction of future research.

Analysis of highly cited references (Table 4) reveals that the knowledge base in this field is composed of three categories of literature. First, the seminal study on ferroptosis (Dixon et al., 2012) first defined this novel form of cell death; it is the most frequently cited and serves as the theoretical foundation for all subsequent research. Second, original discoveries regarding core molecular mechanisms—including the critical role of GPX4 in ferroptosis (Ursini and Maiorino, 2020) and the mechanism by which ACSL4 determines ferroptosis susceptibility through the regulation of lipid composition (Doll et al., 2017)—have provided key targets for understanding the molecular essence of ferroptosis. Finally, a landmark study directly linking ferroptosis to ischemic stroke disease models (Tuo et al., 2017) first validated the role of ferroptosis in cerebral ischemic injury. Subsequent research rapidly delved into stroke-specific mechanisms, such as the protective role of selenium via GPX4 (Alim et al., 2019), ACSL4-mediated inflammation and injury (Cui et al., 2021), and the NCOA4-mediated ferritin autophagy pathway (Li et al., 2021). From the distribution of publications, 2017 marked a significant turning point in this field, after which stroke-related ferroptosis research entered a fast track; Chinese scholars contributed 5 of the 10 highly cited papers, highlighting China’s pivotal role in knowledge construction within this field. Overall, these publications collectively outline a complete chain of knowledge evolution—from conceptual proposal and molecular mechanism elucidation to validation in disease models—laying a solid theoretical foundation for the development of future therapeutic strategies.

**Table 4:** Top 10 co-citation of cited references on “ferroptosis in ischemic stroke” from WOS.

Rank	Title	Type	First author	Source	Publication year	Total citations
1	Tau-mediated iron export prevents ferropototic damage after ischemic stroke (Tuo et al., 2017)	Article	Tuo QZ	Molecular Psychiatry	2017	187
2	Ferroptosis: A Regulated Cell Death Nexus Linking Metabolism, Redox Biology, and Disease (Stockwell et al., 2017)	Review	Stockwell BR	Cell	2017	158
3	ACSL4 exacerbates ischemic stroke by promoting ferroptosis-mediated brain injury and neuroinflammation (Cui et al., 2021)	Article	Cui Y	Brain Behavior and Immunity	2021	151
4	Selenium Drives a Transcriptional Adaptive Program to Block Ferroptosis and Treat Stroke (Alim et al., 2019)	Article	Alim I	Cell	2019	148
5	Ferroptosis: mechanisms, biology and role in disease (Jiang et al., 2021)	Review	Jiang XJ	Nature Reviews Molecular Cell Biology	2021	120
6	ACSLA dictates ferroptosis sensitivity by shaping cellular lipid composition (Doll et al., 2017)	Article	Doll S	Nature Chemical Biology	2017	99
7	Mechanisms of neuronal cell death in ischemic stroke and their therapeutic implications (Tuo et al., 2022)	Review	Tuo QZ	Medicinal Research Reviews	2022	97
8	Nuclear receptor coactivator 4-mediated ferritinophagy contributes to cerebral ischemia-induced ferroptosis in ischemic stroke (Li et al., 2021)	Article	Li C	Nature Chemical Biology	2021	90
9	The neuroprotective effects of carvacrol on ischemia/reperfusion-induced hippocampal neuronal impairment by ferroptosis mitigation (Guan et al., 2019)	Article	Guan XY	Life Science	2019	87
10	Ferroptosis: past, present and future (Li et al., 2020)	Review	Li J	Cell Death &Disease	2020	85

### 3.6 Keyword analysis (primarily based on the WOS database)

**Figure 6:**
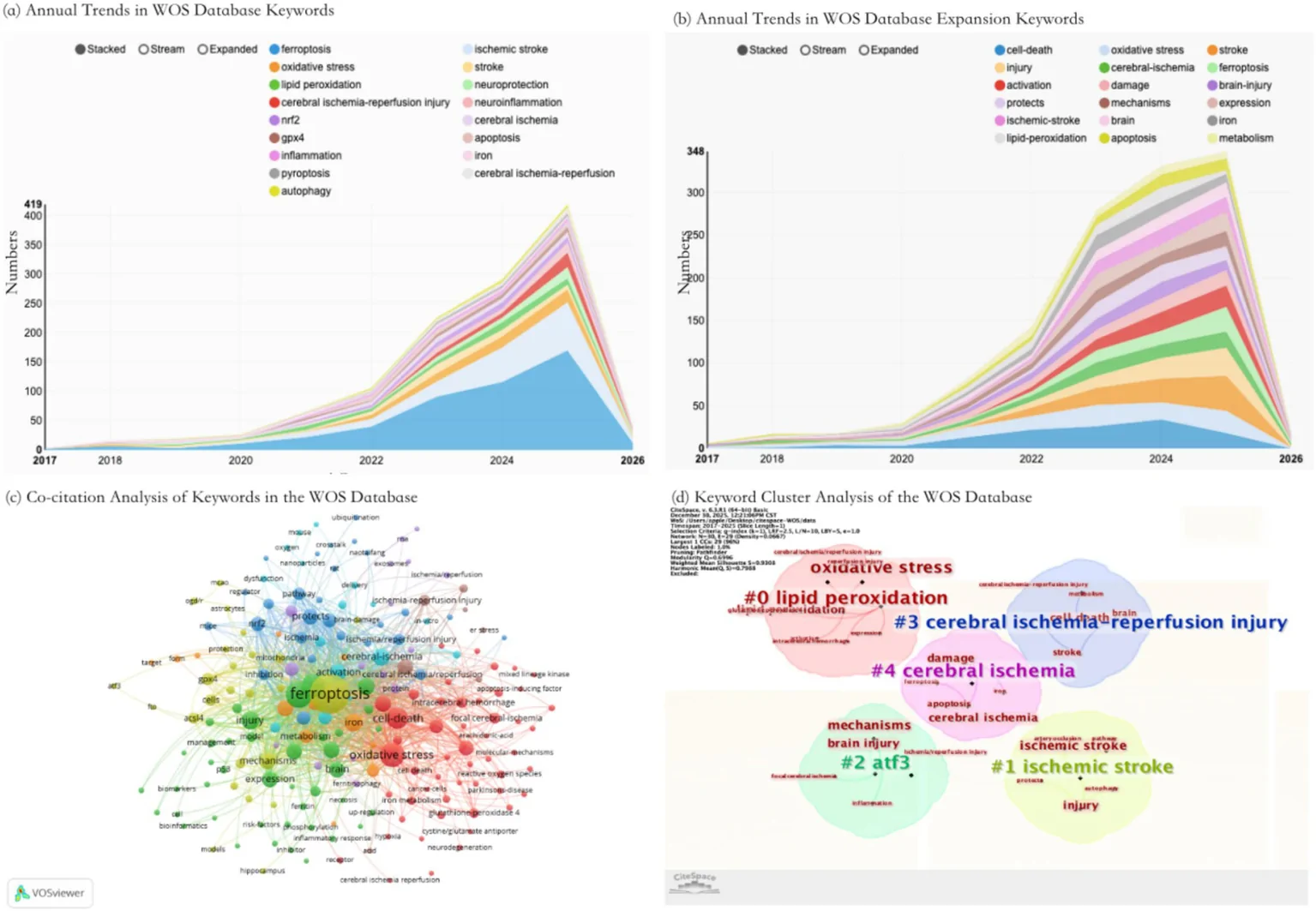
**(a)** Year-by-year changes in keywords in the WOS database. **(b)** Year-by-year changes in expanded keywords in the WOS database. **(c)** Citation analysis of keywords in the WOS database. **(d)** Cluster analysis of keywords in the WOS database.

Based on multidimensional metaanalysis of keywords, this field exhibits a clear knowledge structure and evolutionary patterns. From a temporal perspective (Figure 6a), research hotspots have undergone a complete cycle from conceptual introduction to rapid expansion: the frequency of the term “ferroptosis” has grown exponentially since 2020; “Oxidative stress (Wang et al., 2022)” and “lipid peroxidation” remained central biochemical events throughout; around 2020, keywords related to molecular pathways such as “GPX4” and “Nrf2” rose significantly (Yuan et al., 2021); since 2022, keywords such as “inflammation,” “pyroptosis,” and “autophagy” have rapidly emerged, marking a shift in research focus from single cell death mechanisms to the interaction of multiple death pathways and immune regulatory networks. An expanded keyword analysis (Figure 6b) shows that since 2017, the number of research topics related to this field has continued to increase, with the publication volume for each topic rising in tandem. The overall scale of research peaked in 2024, confirming the ongoing expansion in both the breadth and depth of research, and establishing a multi-directional research landscape centered on ferroptosis, oxidative stress, and ischemic stroke. Co-occurrence analysis of high-frequency keywords (Figure 6c) indicates that the knowledge structure in this field is highly focused on core concepts such as “ferroptosis,” “oxidative stress,” “cell death,” and “damage,” forming a relatively unified cognitive framework. Cluster analysis (Figure 6d) further reveals five major clusters—“lipid peroxidation,” “ischemic stroke,” “cerebral ischemia,” “ferroptosis-apoptosis interaction,” “ATF3,” and “inflammation”—forming a tiered knowledge structure ranging from molecular events, disease models, cell death interactions, and signaling regulation to pathological contexts, thereby comprehensively mapping out the research landscape of this field from mechanistic analysis to the understanding of holistic pathological networks.

### 3.7 Research frontiers and evolving trends: emergent terms and temporal perspectives

**Figure 7:**
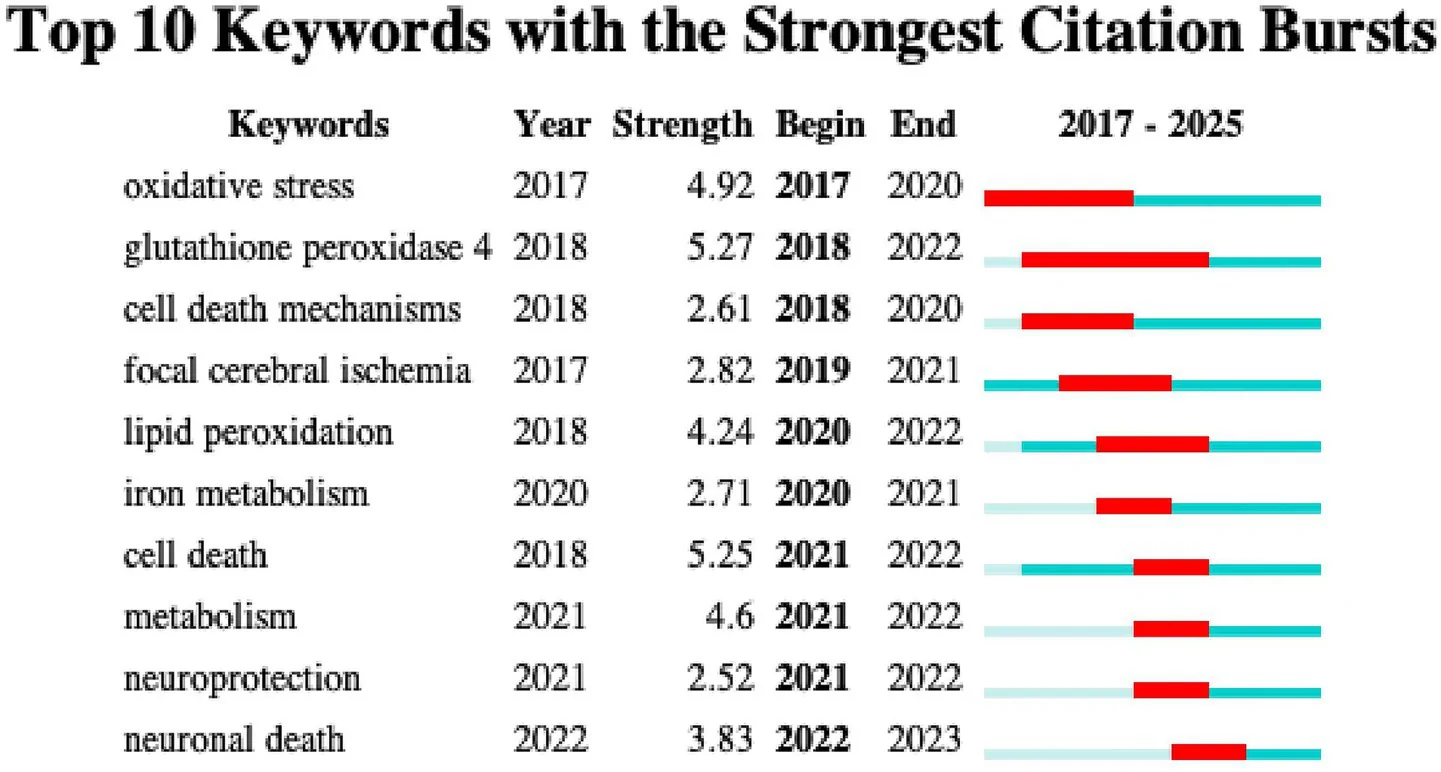
Keyword emergence map, top 10 strengths.

Based on the results of keyword emergence analysis (Figure 7), research hotspots in this field exhibited a clear temporal evolution from 2017 to 2023. From 2017 to 2019, research primarily focused on exploring fundamental mechanisms, with oxidative stress emerging as the initial hotspot, followed by sustained attention on glutathione peroxidase 4 (GPX4) and cell death mechanisms, which laid the theoretical foundation for subsequent studies. From 2020 to 2021, the research focus shifted to specific pathways and disease models, with lipid peroxidation and iron metabolism emerging as core keywords. Concurrently, the widespread application of the focal cerebral ischemia model further propelled mechanism research toward disease-specific contexts. From 2021 to 2023, research hotspots exhibited a trend toward applied translation, cell death once again took center stage, while metabolism and neuroprotection gradually gained prominence. The emergence of “neuronal death” as a new key term signifies that the research perspective has shifted to the mechanisms of cell death in neurological disorders, foreshadowing that future studies will delve deeper into the role of ferroptosis in pathological processes such as cerebral ischemia and neurodegenerative diseases.

**Figure 8:**
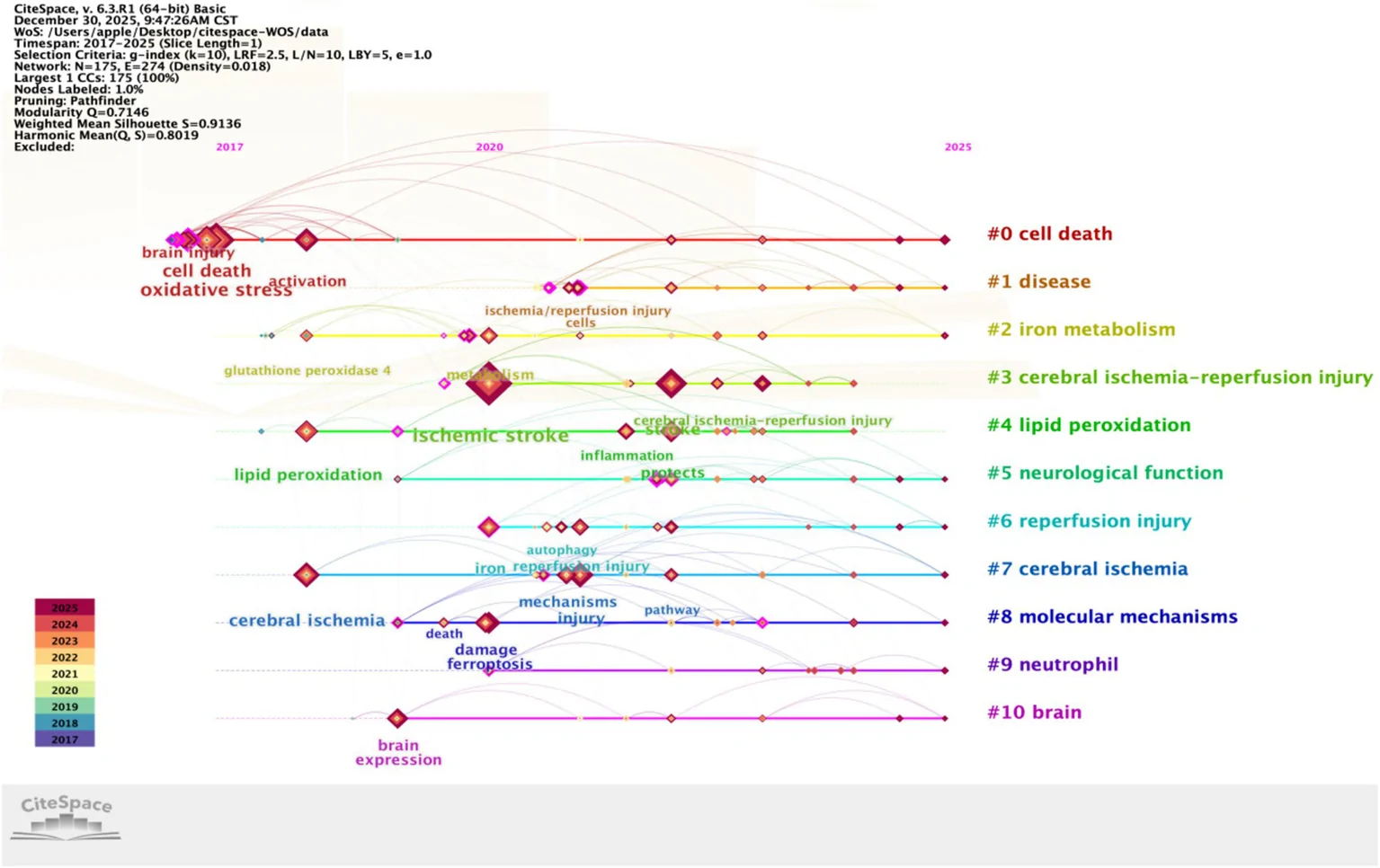
Timezone view.

Based on a visual analysis of the keyword timeline (Figure 8), research in this field exhibits a clear chronological progression and trajectory of knowledge advancement. From a temporal perspective, early research focused on fundamental pathological processes such as “cell death,” “oxidative stress,” and “ischemia–reperfusion injury,” initially establishing the central role of ferroptosis in stroke-induced damage. As research progressed, keywords gradually shifted toward specific molecular mechanisms such as “glutathione peroxidase 4 (GPX4)” and “lipid peroxidation,” marking a refined understanding of the core pathways of ferroptosis. In recent years, research has further expanded to interdisciplinary biological processes such as “autophagy (Liu et al., 2023)” and “inflammation,” reflecting how the complex interplay between ferroptosis, other forms of cell death, and immune regulation has become a new focal point. Overall, this timeline visually illustrates the progressive development of research—from macro-level pathological phenomena to micro-level molecular mechanisms, and ultimately to multi-pathway network regulation—revealing the dynamic process of how the knowledge base in this field continues to expand and mature.

## 4 Discussion

Through systematic bibliometric analysis, this study comprehensively maps the macro-level landscape and developmental trajectory of iron death research in ischemic stroke from 2017 to 2025, revealing the key characteristics of this emerging field as it progresses from basic discoveries to clinical translation.

Compared with the findings of Chen et al. (2021) on stroke in general, our analysis—exclusively focused on ischemic stroke—reveals a marked acceleration in annual publication output after 2022, a shift in keyword clusters toward “ferroptosis-targeted therapy” and “clinical translation,” and a dominant share of Chinese institutions in inhibitor-focused preclinical studies. While Chen et al. noted a lack of international collaboration among Chinese groups in the earlier period, our collaboration network analysis shows that China-US partnerships have since become a central axis in ischemic stroke research.

At first, the field is currently experiencing explosive growth, which is primarily driven by *in vivo* validation of key pathways such as GPX4 and ACSL4, as well as the screening of natural product-based inhibitors. The exponential growth in the number of publications attests to the immense potential of ferroptosis as a new therapeutic target for ischemic stroke and the global consensus surrounding it. China holds an absolute advantage in the number of publications in this field, thanks to the country’s sustained investment in major national strategies such as the National Brain Initiative and precision medicine, as well as the keen insight and efficient execution of domestic research teams in addressing cutting-edge scientific questions. At the same time, the United States, as a traditional scientific and technological powerhouse, remains irreplaceable in terms of its basic research capabilities and role as a global hub. In the future, deepening substantive scientific collaboration between China and the United States—and indeed on a global scale—will be a crucial pathway for facilitating the translation of basic discoveries into clinical applications.

In addition, the knowledge base in this field is solid, and research hotspots are evolving in a progressive manner. Citation analysis indicates that the field is built upon several landmark publications. These include the first demonstration of ferroptosis in stroke (Tuo et al., 2017), the elucidation of ACSL4-mediated lipid peroxidation (Doll et al., 2017; Cui et al., 2021), and the role of ferritinophagy (Li et al., 2021). This publications first established the critical role of ferroptosis in the pathological process of ischemic stroke. Keyword evolution analysis further reveals that the research focus has rapidly expanded from the initial validation of core pathways, such as GPX4 (Cui et al., 2021) and System Xc- (Chen et al., 2025) to upstream signaling regulatory networks, such as Nrf2 (Peng et al., 2024) and ACSL4, which have been thoroughly validated in various disease models, while simultaneously screening a wide range of intervention strategies. This evolutionary trajectory—from molecular to cellular to whole-animal models, and from mechanism elucidation to intervention validation—embodies the classic logic of translational research.

Finally, the research frontier has clearly shifted toward clinical translation and multidimensional mechanistic interactions. Specifically, the keyword “biomarker” (burst since 2023) suggests an urgent need for blood-based ferroptosis markers to stratify stroke patients, while “neurovascular unit” (emerging after 2022) indicates a shift from isolated neuroprotection to multi-cell-type synergy. Analysis of emerging keywords reveals several distinct trends: First of all, translational medicine has become the core orientation. The emergence of terms such as “nanoparticles (Zhao et al., 2024),” “targeted delivery (Yang et al., 2020),” and “biomarkers (Montellano et al., 2021)” indicates that research is striving to overcome bottlenecks in clinical application, focusing on the development of precise delivery systems capable of crossing the blood–brain barrier, as well as specific biomarkers for patient stratification and efficacy assessment. Secondly, mechanism research is becoming more in-depth and interdisciplinary. The emergence of the “neurovascular unit (Wang et al., 2021)” indicates that protective strategies are expanding from single neurons to multi-component synergistic protection involving microvessels and glial cells; meanwhile, the interaction between forms of inflammatory cell death, such as ferroptosis and pyroptosis, has become a new hotspot for elucidating the complex injury network following stroke. Thirdly, drug development is taking on a diversified landscape. In addition to classic ferroptosis inhibitors, innovative drug discovery based on natural products (such as curcumin; Hasanzadeh et al., 2020) and intervention strategies targeting new targets (such as FSP1; Nakamura et al., 2023) are both advancing rapidly.

## 5 Limitations

This study has the following limitations: First, regarding data sources, the analysis included only literature from the Web of Science and PubMed Core databases, failing to cover other international databases such as Scopus and Embase, as well as gray literature resources such as preprints and conference papers, which may have resulted in the omission of some high-quality studies. Second, regarding the search strategy, although it underwent multiple optimizations and pre-search validations, the complex nomenclature and overlapping terminology in ferroptosis research may still result in the failure to fully retrieve some relevant literature. Third, in terms of analytical dimensions, this study did not conduct a systematic review of treatment efficacy; relevant treatment categories were discussed only when they appeared in the bibliometric keyword map. Fourth, regarding methodology, bibliometrics inherently focuses on quantitative analysis and the depiction of macro-level trends, making it difficult to deeply assess the scientific value and innovative contributions of individual papers. An intrinsic evaluation of research quality still requires comprehensive consideration in conjunction with traditional expert reviews and qualitative analysis.

## 6 Conclusion and outlook

Through a bibliometric analysis of stroke-related iron-related death studies in the Web of Science (WoS) database from 2017 to 2025, this study draws the following conclusions: Over the past 8 years, this field has undergone a complete cycle from emergence to rapid growth and has now become one of the most active frontier areas in neuroscience and stroke research. The research trajectory is clear: it has evolved from the introduction of the concept and exploration of core mechanisms to the comprehensive mapping of regulatory networks, and is now advancing toward a new phase of clinical translation and cross-mechanism integration. Global collaboration is key to driving quality improvement.

Looking ahead, research on ferroptosis in ischemic stroke will continue to deepen in the following directions: (1) Refinement of mechanism exploration: Utilizing ACSL4-focused multi-omics and ferritinophagy-specific transcriptomics to elucidate the dynamic patterns of ferroptosis at single-cell resolution across different cell types, brain regions, and disease time points. The sustained burst strength of “ACSL4” (2021–2025) and “ferritinophagy” (2022–2025) indicates that these two pathways should be prioritized for future mechanistic studies. (2) Innovation in technological applications: Developing more advanced organoid models, *in vivo* imaging techniques, and gene editing tools to study the ferroptosis process in real time and visually. (3) Accelerated clinical translation: Promoting targeted nanomedicines against ACSL4 and small-molecule inhibitors of ferritinophagy (e.g., NCOA4 inhibitors) and novel small-molecule inhibitors with proprietary intellectual property rights into clinical trials; establishing a multimodal biomarker system based on blood tests, imaging, and biomarker development based on peripheral lipid peroxidation products (4-HNE, malondialdehyde). (4) Integrated therapeutic strategies: Exploring synergistic effects between ferroptosis inhibitors and existing or emerging therapies such as thrombolysis, thrombectomy, anti-inflammation, and neurogenesis, to construct a personalized, combination-based comprehensive treatment system.

In summary, bibliometric analysis provides both a “telescope” and a “microscope” to survey this field. Ferroptosis research offers a new perspective and arsenal for combating ischemic stroke, a major disease. Its future development is highly anticipated and is expected to ultimately bring hope to millions of stroke patients worldwide.
